# Comparison Between Urothelial and Non-Urothelial Urethral Cancer

**DOI:** 10.3389/fonc.2020.629692

**Published:** 2021-01-29

**Authors:** Mike Wenzel, Marina Deuker, Luigi Nocera, Claudia Collà Ruvolo, Zhe Tian, Shahrokh F. Shariat, Fred Saad, Alberto Briganti, Andreas Becker, Luis A. Kluth, Felix K.H. Chun, Pierre I. Karakiewicz

**Affiliations:** ^1^Department of Urology, University Hospital Frankfurt, Goethe University Frankfurt, Frankfurt am Main, Germany; ^2^Cancer Prognostics and Health Outcomes Unit, Division of Urology, University of Montréal Health Center, Montréal, QC, Canada; ^3^Department of Urology and Division of Experimental Oncology, URI, Urological Research Institute, IBCAS San Raffaele Scientific Institute, Milan, Italy; ^4^Department of Neurosciences, Reproductive Sciences and Odontostomatology, University of Naples Federico II, Naples, Italy; ^5^Department of Urology, Comprehensive Cancer Center, Medical University of Vienna, Vienna, Austria; ^6^Departments of Urology, Weill Cornell Medical College, New York, NY, United States; ^7^Department of Urology, University of Texas Southwestern, Dallas, TX, United States; ^8^Department of Urology, Second Faculty of Medicine, Charles University, Prague, Czechia; ^9^Institute for Urology and Reproductive Health, I.M. Sechenov First Moscow State Medical University, Moscow, Russia; ^10^Division of Urology, Department of Special Surgery, Jordan University Hospital, The University of Jordan, Amman, Jordan

**Keywords:** adenocarcinoma, chemotherapy, metastatic urethral cancer, mortality, squamous cell carcinoma, variant histology, urethral cancer, non-urothelial

## Abstract

**Background:**

To test the effect of variant histology relative to urothelial histology on stage at presentation, cancer specific mortality (CSM), and overall mortality (OM) after chemotherapy use, in urethral cancer.

**Materials and Methods:**

Within the Surveillance, Epidemiology and End Results (2004–2016) database, we identified 1,907 primary variant histology urethral cancer patients. Kaplan-Meier plots, Cox regression analyses, cumulative incidence-plots, multivariable competing-risks regression models and propensity score matching for patient and tumor characteristics were used.

**Results:**

Of 1,907 eligible urethral cancer patients, urothelial histology affected 1,009 (52.9%) vs. squamous cell carcinoma (SCC) 455 (23.6%) vs. adenocarcinoma 278 (14.6%) vs. other histology 165 (8.7%) patients. Urothelial histological patients exhibited lower stages at presentation than SCC, adenocarcinoma or other histology patients. In urothelial histology patients, five-year CSM was 23.5% vs. 34.4% in SCC [Hazard Ratio (HR) 1.57] vs. 40.7% in adenocarcinoma (HR 1.69) vs. 43.4% in other histology (HR 1.99, p < 0.001). After matching in multivariate competing-risks regression models, variant histology exhibited 1.35-fold higher CSM than urothelial. Finally, in metastatic urethral cancer, lower OM was recorded after chemotherapy in general, including metastatic adenocarcinoma and other variant histology subtypes, except metastatic SCC.

**Conclusion:**

Adenocarcinoma, SCC and other histology subtypes affect fewer patients than urothelial histology. Presence of variant histology results in higher CSM. Finally, chemotherapy for metastatic urethral cancer improves survival in adenocarcinoma and other variant histology subtypes, but not in SCC.

## Introduction

Primary urethral cancer is a rare urological tumor with an incidence of 4.3 and 1.5 per million for men and women, respectively (1). Urothelial histology represents the predominant histological subtype in urethral cancer. It is followed by variant histologies that include squamous cell carcinoma (SCC), adenocarcinoma and other histological subtypes such as Mullerian type, melanocytic, neuroendocrine, mesenchymal, sarcoma, and spindle cell entities (2, 3).

Urethral cancer has been studied in small patient groups and several studies were based on patients diagnosed in the 1970s and 1980s (1, 4–7). In those studies, variant histology predisposed to more advanced stage and worse survival. However, this association was not tested in contemporary urethral cancer patients. Similarly, the effect of chemotherapy was not thoroughly tested in metastatic urethral cancer patients, especially after stratifying according to histological subtype (8, 9).

In consequence, we addressed these two points and relied on the Surveillance, Epidemiology and End Results (SEER) database (2004–2016). We hypothesized that histological subtype may affect stage at presentation, response to chemotherapy and survival. Specifically, we tested the effect of histological subtype (urothelial vs. squamous cell carcinoma vs. adenocarcinoma vs. other histology) on stage at presentation, as well as on cancer specific mortality (CSM). Moreover, we tested the effect of chemotherapy on overall mortality (OM), according to histological subtype in metastatic urethral cancer patients.

## Materials and Methods

### Study Population

The current SEER database samples 34.6% of the United States population and approximates it in demographic composition and cancer incidence (10). Within SEER database (2004−2016), we identified patients ≥18 years old with histologically confirmed urethral cancer [International Classification of Disease for Oncology (ICD-O) site code C68.0]. Histological subtype was defined as either urothelial, SCC, adenocarcinoma, or other histologies. according to WHO criteria (11). Unknown histology was excluded. Cases identified only at autopsy or death certificate were excluded. TNM-stage was used according the 8th edition of malignant tumors (12). According to SEER mortality code, CSM was defined as deaths related to urethral cancer. All other deaths were considered as other cause mortality (OCM).

All analyses and their reporting followed the SEER reporting guidelines. Due to the anonymously coded design of the SEER database, study-specific Institutional Review Board ethics approval was not required.

### Statistical Analysis

Descriptive statistics included frequencies and proportions for categorical variables. Means, medians, and interquartile-ranges were reported for continuously coded variables. The Chi-square tested the statistical significance in proportions’ differences. The t-test and Kruskal-Wallis test examined the statistical significance of means’ and distributions’ differences. Trend tests were performed to explore differences and according to stage at presentation.

Using propensity score matching, CSM was adjusted for OCM. Propensity score matching was performed for comparisons between histology subtypes, according to age at diagnosis (interval: ≤ 2 years), T-stage (T1-2 vs. T3-4), N-stage (N0/Nx vs. N+) and M-stage (M0/Mx vs. M1). The endpoint of interest in survival analyses that relied on non-metastatic patients, consisted of CSM and was addressed in cumulative incidence plots and in univariable, as well as multivariable competing-risks regression (CRR) models. The objective was to maximally reduce those differences with the intent of illustrating CSM, in a fashion that minimizes the contribution of any other variable, except for histological subtype. Since propensity score matching only allows comparisons between two groups, we performed four different sets of separate comparisons addressing CSM. First, we tested for CSM differences between for urothelial vs. non-urothelial histological subtype. Second, we tested for CSM differences between urothelial vs. SCC. Third, we tested for CSM differences between for urothelial vs. adenocarcinoma. Fourth, we tested for CSM differences between for urothelial vs. other histology.

Finally, in metastatic urethral cancer, Kaplan-Meier plots and multivariable cox regression models were fitted to test effects of chemotherapy on OM in the overall cohort, as well as in histological subtype specific subgroups. All tests were two sided with a level of significance set at p <0.05 and R software environment for statistical computing and graphics (version 3.4.3, Boston, United States) was used for all analyses.

## Results

### Descriptive Characteristics of the Study Population

The applied selection criteria yielded to 1,907 urethral cancer patients of whom 181 harbored metastatic urethral cancer at diagnosis. In the overall cohort, urothelial histological subtype affected 1,009 (52.9%) vs. SCC 455 (23.6%) vs. adenocarcinoma 278 (14.6%) vs. other histological subtype 165 (8.7%) patients, respectively (**Table 1**). Of all, 1302 (68.3%) were male. Median age at diagnosis was higher in urothelial histological subtype (75 years) vs. SCC (66 years) vs. adenocarcinoma (69 years) vs. other histological subtype (70 years), respectively (p < 0.001). In 181 metastatic urethral cancer patients, urothelial histology subtype affected 90 (49.7%) vs. SCC 38 (21.0%) vs. adenocarcinoma 33 (18.2%) vs. other histology 20 (11.0%) patients.

**Table 1:** Characteristics of urothelial vs. non-urothelial urethral cancer patients.

Variable		Overall N = 1,907	UrothelialN = 1,009 (52.9%)	SCCN = 455 (23.9%)	AdenocarcinomaN = 278 (14.6%)	OtherN = 165 (8.7%)	P value
**Age at diagnosis**	Median (IQR)	72 (62–81)	75 (66–82)	66 (56–77)	69 (58–78)	70 (59–80)	<0.001
**Gender**	Female	605 (31.7)	174 (17.2)	149 (32.7)	166 (59.7)	116 (70.3)	<0.001
	Male	1,302 (68.3)	835 (82.8)	306 (67.3)	112 (40.3)	49 (29.7)	
**Race/ethnicity**	Caucasian	1,356 (71.1)	791 (78.4)	315 (69.2)	157 (56.5)	93 (56.4)	<0.001
	African-American	312 (16.4)	101 (10)	90 (19.8)	79 (28.4)	42 (25.5)	
	Hispanic	134 (7.0)	71 (7.0)	27 (5.9)	20 (7.2)	16 (9.7)	
	Other	102 (5.3)	46 (4.6)	21 (4.6)	22 (7.9)	13 (7.9)	
**T stage**	T1	694 (36.4)	412 (40.8)	160 (35.2)	82 (29.5)	40 (24.2)	<0.001
	T2	168 (8.8)	115 (11.4)	24 (5.3)	17 (6.1)	12 (7.3)	
	T3	625 (32.8)	298 (29.5)	176 (38.7)	98 (35.3)	53 (32.1)	
	T4	216 (11.3)	102 (10.1)	50 (11)	40 (14.4)	24 (14.5)	
	Tx/Unknown	204 (10.7)	82 (8.1)	45 (9.9)	41 (14.7)	36 (21.8)	
**N stage**	N0/Nx	1,570 (82.3)	865 (85.7)	343 (75.4)	226 (81.3)	136 (82.4)	<0.001
	N+	337 (17.7)	144 (14.3)	112 (24.6)	52 (18.7)	29 (17.6)	
**M Stage**	M0/Mx	1,726 (90.5)	919 (91.1)	417 (91.6)	245 (88.1)	145 (87.9)	<0.001
	M1	181 (9.5)	90 (8.9)	38 (8.4)	33 (11.9)	20 (12.1)	
**Treatment**	Endoscopic/Surgery	941 (49.3)	542 (53.7)	200 (44)	118 (42.4)	81 (49.1)	<0.001
	Bi/Trimodality	503 (26.4)	267 (26.5)	114 (25.1)	76 (27.3)	46 (27.9)	
	Systemic chemotherapy	184 (9.6)	66 (6.5)	69 (15.2)	31 (11.2)	18 (10.9)	
	Unknown	279 (14.6)	134 (13.3)	72 (15.8)	53 (19.1)	20 (12.1)	

Descriptive characteristics of 1,907 urethral cancer patients, stratified according to histologies, namely urothelial, squamous cell carcinoma, adenocarcinoma and other, diagnosed within the Surveillance, Epidemiology, and End Results database from 2004 to 2016. SCC, Squamous cell carcinoma; IQR, Inter quartile range.

### The Effect of Variant Histologies on Stage at Presentation

**Figure 1:**
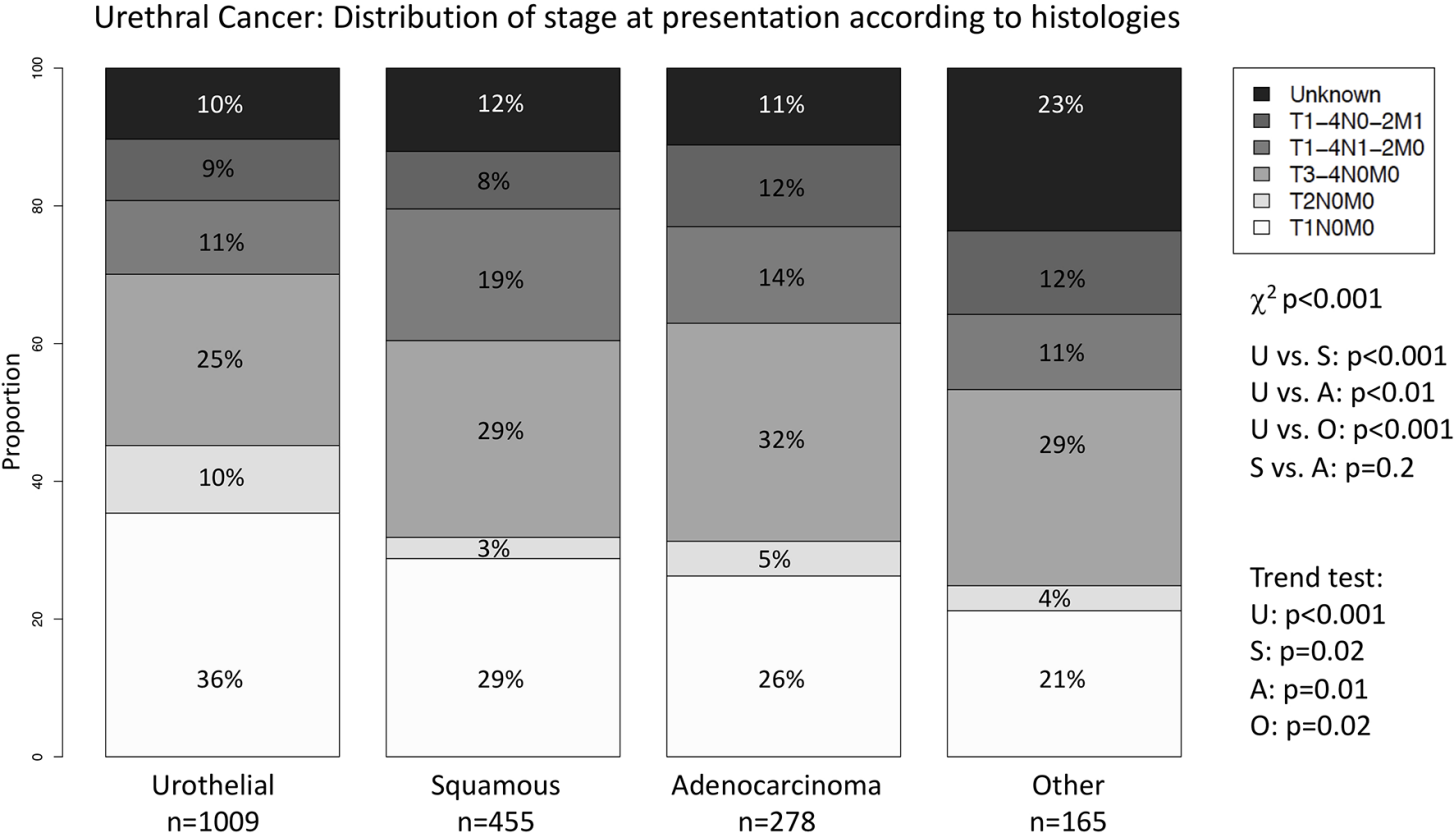
Stage distribution according to histological subtype in urethral cancer. Stacked barplots depicting the stage at presentation according to histological subtype in 1,907 urethral cancer patients. U, Urothelial; S, Squamous Cell Carcinoma; A, Adenocarcinoma; O, Other; χ^2^, Chi-Square.

In general, we recorded differences in stage at presentation between urothelial vs. variant histology patients (p < 0.01; **Figure 1**). Conversely, no differences were recorded between SCC and adenocarcinoma patients (p = 0.2). For example, the highest proportion of T1N0M0 stage was recorded in urothelial histology subtype patients (n = 357; 36%) vs. SCC (n = 131; 29%), adenocarcinoma (n = 73; 26%), and other histologies (n = 35, 21%). Conversely, the most frequent stage in SCC (n = 130, 29%), adenocarcinoma (n = 88; 32%), and other histology (n = 47; 29%) was T3-4N0M0, but not in urothelial histology subtype (n = 251; 25%). No clinically meaningful differences were recorded according to histological subtype in N1-2 or M1 patients. Highest proportion of advanced stages was recorded in SCC histology subtype followed by adenocarcinoma, followed by other histology and urothelial histology subtype (trend test all p ≤ 0.02).

### The Effect of Variant Histologies on CSM Before and After Propensity Score Matching

**Figure 2:**
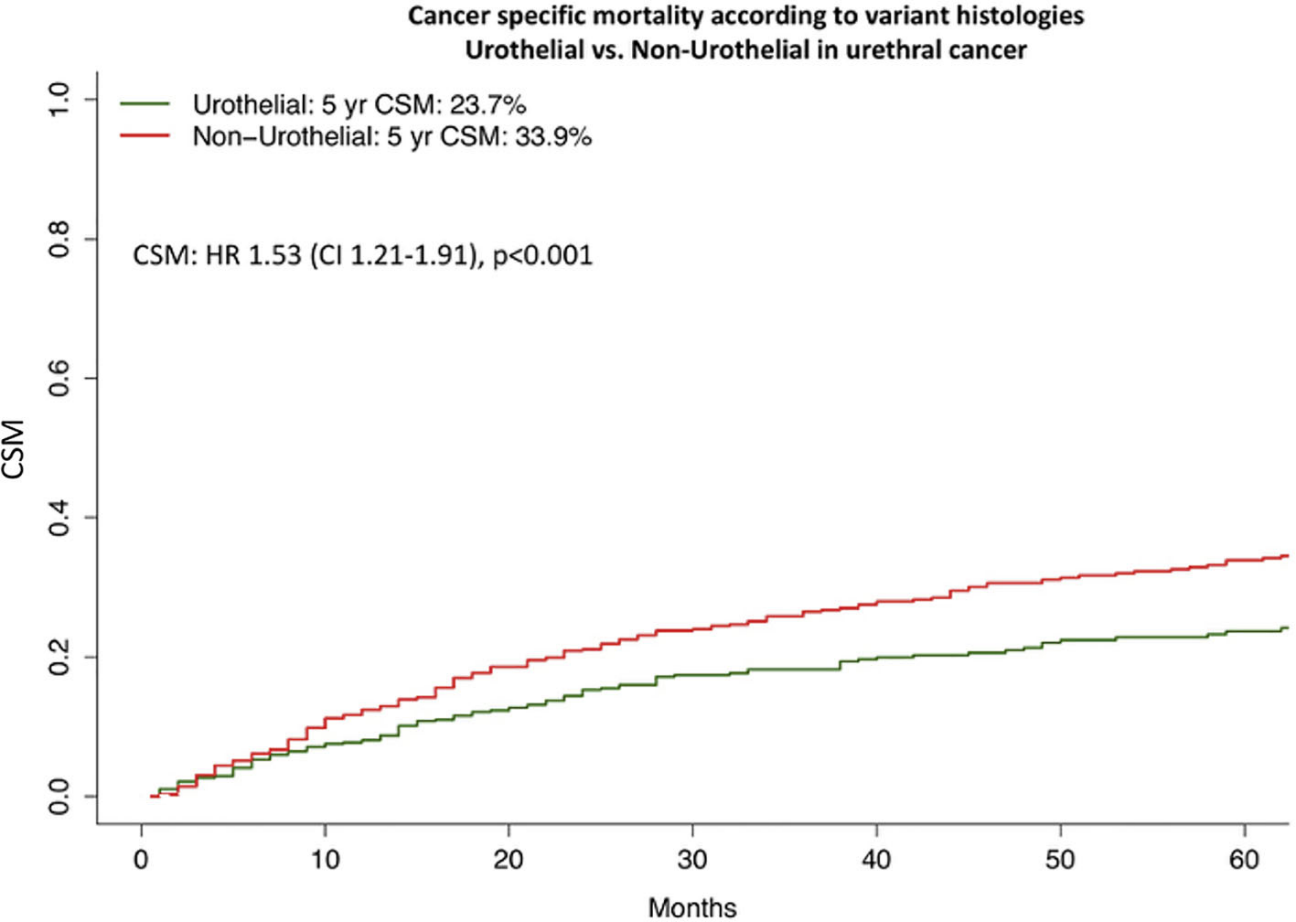
Cancer specific mortality of urothelial vs. non-urothelial histology in urethral cancer. Cumulative incidence plots illustrating cancer specific mortality (CSM) after 1:1 propensity score matching for TNM-stage and age at diagnoses in urothelial vs. non-urothelial histology urethral cancer patients (both n = 765). HR, Hazard Ratio; CI, Confidence Interval.

**Figure 3:**
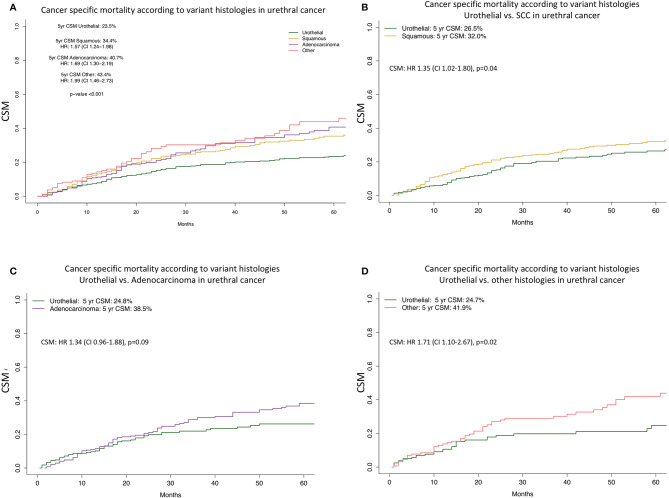
Cancer specific mortality of urothelial vs. variant histologies in urethral cancer. Cumulative incidence plots illustrating cancer specific mortality (CSM) before **(A)** and after 1:1 propensity score matching for TNM-stage, age **(B–D)**. Panel **(A)** shows the unmatched comparison between urothelial (n = 1,009), SCC (n = 455), adenocarcinoma (n = 278), and other histology (n = 165). Panel **(B)** depicted the matched comparison between urothelial and SCC (both n = 435). Panel **(C)** depicted the matched comparison between urothelial and adenocarcinoma (both n = 278). Panel **(D)** depticted the matched comparison between urothelial and other histology (both n = 163). HR, Hazard ratio; CI, Confidence interval; CSM, Cancer specific mortality; yr, year.

CSM analysis according to histology subtype demonstrated important differences (**Figures 2**, **3**). Specifically, five-year CSM was 23.5% in urothelial histology vs. 34.4% in SCC [Hazard Ratio (HR) 1.57] vs. 40.7% in adenocarcinoma (HR 1.69) vs. 43.4% in other histology subtype patients (HR 1.99, p < 0.001).

Due to important differences in stage at presentation and patient age, we relied on matching to test for CSM differences between urothelial vs. variant histology (**Figures 2**, **3**). The objective was to maximally reduce those differences with the intent of illustrating CSM, in a fashion that minimizes the contribution of any other variable, except for histological subtype. Prior to matching, 1,009 urothelial histology and 455 SCC, 278 adenocarcinoma and 165 other histology patients were available for analyses. Several comparisons were performed. The first comparison focused on urothelial vs. variant histology patients and relied on 765 urothelial vs. 765 variant histology patients after 1:1 propensity score matching for stage and age. After additional multivariate adjustment in matched analyses for race/ethnicity and treatment type revealed a 1.35-fold higher CSM (p = 0.01) for variant histology. In the second comparison after matching of 278 urothelial vs. 278 SCC patients and after further multivariate adjustment, the analyses revealed a 1.17-fold higher CSM (p = 0.3) for SCC (**Table 2**). In the third comparison after matching of 435 urothelial vs. 435 adenocarcinoma patients and after further multivariate adjustment, the analyses revealed virtually no CSM difference (HR 1.05, p = 0.8). In the fourth comparison of 163 urothelial vs. 163 other variant histology patients after matching and after further multivariate adjustment, the analyses revealed a 1.68-fold higher CSM (p = 0.03) for other histology (**Table 3**).

**Table 2:** Univariable and multivariable competing-risks regression models for urethral cancer patients (Urothelial vs. Variant histology/Urothelial vs. SCC).

	CSM Urothelial vs. Variant histology				CSM Urothelial vs. SCC			
	Univariable		Multivariable		Univariable		Multivariable	
	HR (95% CI)	P-value	HR (95% CI)	P-value	HR (95% CI)	P-value	HR (95% CI)	P-value
**Histology**								
Urothelial	1.00 (Ref.)	---	1.00 (Ref.)	---	1.00 (Ref.)	---	1.00 (Ref.)	---
Variant histology/SCC	1.53 (1.23–1.91)	<0.001	1.35 (1.07–1.69)	0.01	1.35 (1.02–1.80)	0.036	1.17 (0.87–1.59)	0.3
**Race/ethnicity**								
Caucasian	1.00 (Ref.)	---	1.00 (Ref.)	---	1.00 (Ref.)	---	1.00 (Ref.)	---
African American	1.80 (1.39–2.32)	<0.001	1.64 (1.26–2.12)	<0.001	1.60 (1.12–2.28)	<0.01	1.44 (1.01–2.07)	0.045
Hispanic	1.31 (0.85–2.00)	0.2	1.28 (0.83–1.97)	0.3	1.26 (0.72–2.21)	0.4	1.4 (0.8–2.44)	0.2
Other	1.58 (1.04–2.40)	0.03	1.45 (0.95–2.21)	0.09	1.54 (0.82–2.87)	0.18	1.33 (0.71–2.51)	0.4
**Treatment**								
Endoscopic/Surgery	1.00 (Ref.)	---	1.00 (Ref.)	---	1.00 (Ref.)	---	1.00 (Ref.)	---
Bi-/Trimodality	1.65 (1.27–2.15)	<0.001	1.67 (1.28–2.18)	<0.001	2.44 (1.71–3.49)	<0.001	2.47 (1.72–3.55)	<0.001
Systemic chemotherapy	3.01 (2.18–4.16)	<0.001	2.89 (2.09–3.99)	<0.001	4.24 (2.77–6.49)	<0.001	4.06 (2.61–6.29)	<0.001
Unknown	2.70 (1.96–3.71)	<0.001	2.62 (1.9–3.61)	<0.001	3.54 (2.28–5.51)	<0.001	3.38 (2.17–5.26)	<0.001

Univariable and multivariable competing-risks regression models for urethral cancer patients matched for TNM-stage and age for urothelial vs. variant histology and subsequently for urothelial vs. squamous cell carcinoma.

CSM, Cancer specific mortality; SCC, Squamous cell carcinoma; HR, Hazard ratio; CI, Confidence interval.

**Table 3:** Univariable and multivariable competing-risks regression models for urethral cancer patients (Urothelial vs. Adenocarcinoma/Urothelial vs. Other variant histology).

	CSM Urothelial vs. Adenocarcinoma				CSM Urothelial vs. Other variant histology			
	Univariable		Multivariable		Univariable		Multivariable	
	HR (95% CI)	P-value	HR (95% CI)	P-value	HR (95% CI)	P-value	HR (95% CI)	P-value
**Histology**								
Urothelial	1.00 (Ref.)	---	1.00 (Ref.)	---	1.00 (Ref.)	---	1.00 (Ref.)	---
Adenocarcinoma/Other variant histology	1.34 (0.96–1.88)	0.09	1.05 (0.73–1.49)	0.3	1.71 (1.10–2.67)	0.02	1.68 (1.06–2.68)	0.03
**Race/ethnicity**								
Caucasian	1.00 (Ref.)	---	1.00 (Ref.)	---	1.00 (Ref.)	---	1.00 (Ref.)	---
African American	2.11 (1.46–3.04)	<0.001	1.83 (1.26–2.66)	0.045	1.73 (1.06–2.83)	0.03	1.49 (0.91–2.43)	0.11
Hispanic	1.27 (0.62–2.58)	0.5	1.22 (0.58–2.56)	0.2	1.07 (0.45–2.54)	0.9	0.95 (0.38–2.38)	0.9
Other	1.45 (0.72–2.93)	0.3	1.12 (0.51–2.46)	0.4	1.30 (0.57–2.97)	0.5	1.23 (0.57–2.68)	0.6
**Treatment**								
Endoscopic/Surgery	1.00 (Ref.)	---	1.00 (Ref.)	---	1.00 (Ref.)	---	1.00 (Ref.)	---
Bi-/Trimodality	2.06 (1.33–3.18)	<0.01	2.05 (1.33–3.15)	<0.001	1.51 (0.89–2.55)	0.13	1.50 (0.88–2.54)	0.14
Systemic chemotherapy	5.93 (3.69–9.54)	<0.001	5.45 (3.33–8.94)	<0.001	2.57 (1.34–4.91)	<0.01	2.42 (1.29–4.53)	<0.001
Unknown	3.21 (1.90–5.44)	<0.001	3.09 (1.79–5.35)	<0.001	2.94 (1.52–5.66)	<0.01	3.10 (1.63–5.9)	<0.001

Univariable and multivariable competing-risks regression models for urethral cancer patients matched for TNM-stage and age for urothelial vs. adenocarcinoma and subsequently for urothelial vs. other histology. CSM, Cancer specific mortality; HR, Hazard ratio; CI, Confidence interval.

### The Effect of Systemic Chemotherapy in Metastatic Urethral Cancer According to Variant Histologies

**Figure 4:**
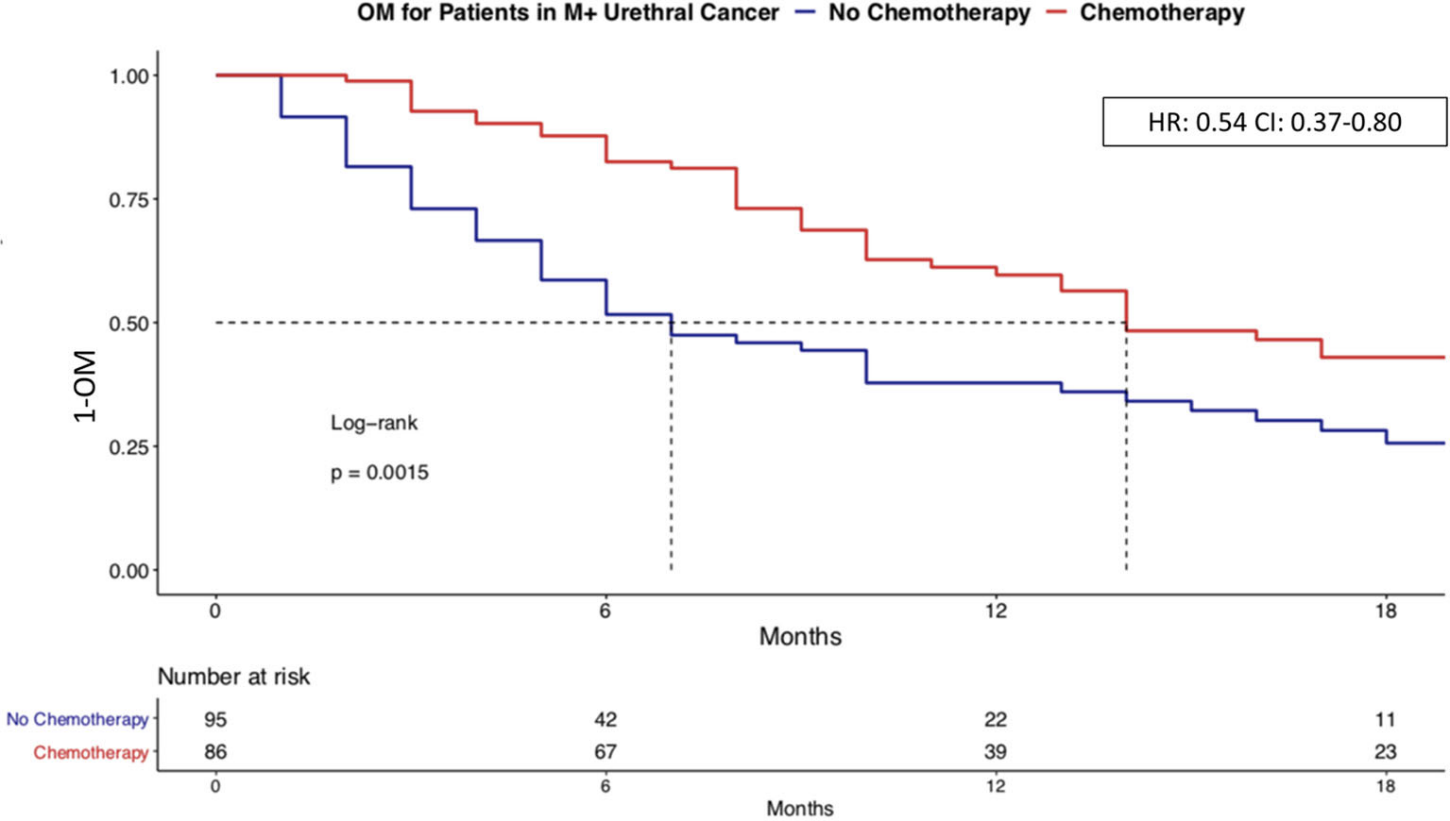
Overall mortality of chemotherapy vs. no chemotherapy in metastatic urethral cancer. Kaplan-Meier plot illustrating overall mortality (OM) for metastatic urethral cancer within comparing chemotherapy (n = 86) vs. no chemotherapy (n = 95). OM, Overall mortality; HR, Hazard ratio; CI, Confidence interval.

In the subgroup of 181 metastatic urethral cancer patients, we first tested for the effect of chemotherapy on OM across all histological variants, without stratification between urothelial vs. variant histology. Here, chemotherapy use resulted in median survival of 14 vs. 7 months without chemotherapy use (HR: 0.54, p < 0.01). After multivariable adjustment for age, race/ethnicity, patient sex and treatment type, chemotherapy use was an independent predictor of lower OM (HR 0.47, p < 0.01; **Figure 4**).

**Figure 5:**
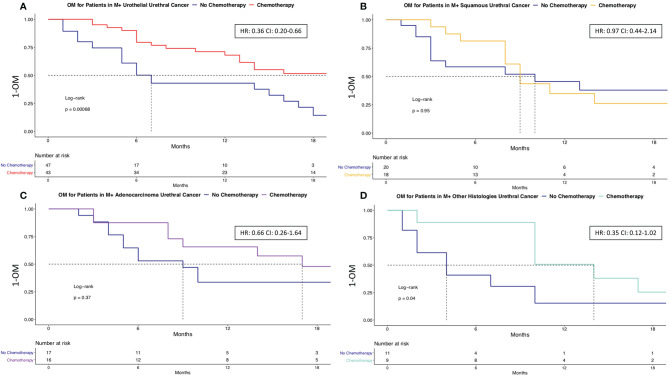
Overall mortality of chemotherapy vs. no chemotherapy in variant histologies of metastatic urethral cancer. Kaplan-Meier plots illustrating overall mortality (OM) in metastatic urethral cancer patients for urothelial and variant histology. Panel **(A)** depicted the comparison between chemotherapy and no chemotherapy in urothelial histological subtype. Panel **(B)** depicted the comparison between chemotherapy and no chemotherapy in squamous cell carcinoma histological subtype. Panel **(C)** depicted the comparison between chemotherapy and no chemotherapy in adenocarcinoma histological subtype. Panel **(D)** depicted the comparison between chemotherapy and no chemotherapy in other histological subtypes. OM, Overall mortality; HR, Hazard ratio; CI, Confidence interval; Squamous, Squamous cell carcinoma.

Subsequently, we repeated the OM analyses in four subtype-specific analyses: 1) adenocarcinoma, 2) other variant histology 3) SCC, finally in 4) urothelial histology. In the analysis addressing chemotherapy use in metastatic adenocarcinoma histology, chemotherapy was associated with lower OM in univariate analyses (HR 0.66, p = 0.6) as well as after further multivariate adjustment for age, race/ethnicity, and patient sex (HR 0.19, p = 0.11). In the analysis addressing chemotherapy use in metastatic other variant histology (**Figure 5**), chemotherapy was associated with lower OM in univariate analyses (HR 0.35, p = 0.04) as well as after further multivariate adjustment for age, race/ethnicity, and patient sex (HR 0.36, p = 0.068). In the analysis addressing chemotherapy use in metastatic urothelial histology, chemotherapy was associated with lower OM in univariate analyses (HR 0.29, p = 0.038). In metastatic SCC histology, chemotherapy use did not affect OM in either univariate (HR: 0.97, p = 0.9) or multivariate analyses (HR: 2.93, p = 0.4).

## Discussion

We hypothesized that histological subtype may affect stage at presentation, response to chemotherapy and survival. We tested this hypothesis within the SEER database and arrived at several important observations.

First, we identified important differences in stage distribution according to histological subtype. Specifically, variant histology was associated with higher proportion of locally advanced stage than urothelial histology subtype. Moreover, within variant histology, SCC and other histology subtype demonstrated a more unfavorable stage distribution pattern than urothelial histology. Finally, adenocarcinoma demonstrated the worse predisposition to locally advanced stage at diagnoses. Taken together, these observations indicate that in general variant histology predispose to worse stage at presentation than urothelial histology. Moreover, within variant histology, SCC stage distribution was the closest to urothelial. Conversely, adenocarcinoma and other variant histology subgroup showed the most pronounced stage difference relative to urothelial and the highest rates of locally advanced tumors. To the best of our knowledge, we are the first to report the observed differences between urothelial vs. variant histology as well as the differences in stage distribution that exist within the three variant histology groups examined in this study. For example, previous investigators based their conclusions on very small patient groups (n = 91), relative to the current study (6). In consequence, the robustness of their findings and conclusions was limited.

Second, we observed important differences in stage and age distribution according to histological subtypes. Based on these differences we relied on propensity score matching. The objective of matching was to maximally adjust for age and stage differences with the intent of illustrating the most unbiased and the most direct effect of variant histology on CSM, relative to urothelial histology. In addition to propensity score matching, we also relied on additional multivariable adjustment for residual variables, namely race/ethnicity and treatment. Finally, based on methodological considerations for matched comparisons between two groups, we first applied the above steps to the comparison between urothelial vs. variant histology, in general. Subsequently, we repeated the analyses in variant histology-specific comparison: 1) SCC vs. urothelial, 2) adenocarcinoma vs. urothelial, 3) other variant histology vs. urothelial.

The comparison between urothelial vs. variant histology revealed higher CSM in variant histology. Subsequently, the three pairwise comparisons between SCC vs. urothelial, adenocarcinoma vs. urothelial, other variant histology vs. urothelial corroborated a CSM disadvantage of SCC and other variant histology in univariable competing risk regression models (SCC: HR 1.35, other variant histology: HR 1.71, both p < 0.05), but only for other variant histologies in multivariable analyses. In the comparison between adenocarcinoma vs. urothelial, higher CSM was also recorded for adenocarcinoma, albeit in a statistically non-significant fashion (HR 1.34, p = 0.09). It is noteworthy that magnitude of the CSM difference between adenocarcinoma and urothelial was highly comparable to the one recorded in the comparison that relied on variant histology in general, as well as in SCC and other variant histology subgroups. Taken together, these observations indicate that even after matching and most detailed adjustment for stage and age, variant histology patients fare significantly worse than their urothelial counterparts. It is noteworthy that the magnitude of this disadvantage was highly comparable across all three variant histology subgroups. It should be emphasized that the survival disadvantage was recorded despite 1:1 matching that eliminated the stage disadvantage, associated with variant histology, especially in other variant histology, where the disadvantaged was the strongest. To the best of our knowledge, no previous investigators focused on that topic. In consequence, our findings cannot be compared with other studies. Nonetheless, the results should be considered in clinical management of urethral cancer patients.

Third, we also focused on a subgroup of 181 metastatic urethral cancer patients. Here, we tested for chemotherapy effect on OM in urethral cancer variant histology patients, since no previous study examined this concept. Our analyses revealed a decrease in OM with chemotherapy use in metastatic adenocarcinoma, metastatic other variant histology, as well as metastatic urothelial histology, but not in metastatic SCC. To the best of our knowledge, we are the first to systematically examine the effect of chemotherapy use in metastatic urethral cancer. In consequence, we cannot make direct comparisons with previous studies. However, our findings are consistent with the known effect of chemotherapy in bladder cancer, where systemic chemotherapy is also associated with a survival benefit in metastatic urothelial, metastatic adenocarcinoma, and metastatic neuroendocrine but not in metastatic SCC (13, 14).

In summary, variant histology is associated with less favorable stage distribution at diagnosis. Similarly, variant histology is associated with higher CSM relative to urothelial histology. Within all variant histology patients, CSM differences in SCC and other variant histology existed, where the highest CSM was recorded. Finally, in metastatic variant histology urethral cancer patients, we demonstrated that chemotherapy exposure reduced OM in adenocarcinoma and other variant histology, but not in SCC. These findings indicate that more advanced stages at presentation should be expected in variant histology urethral cancer patients. Moreover, despite holding stage and other characteristics constant, clinicians should expect higher mortality across all variant histology stages, relative to urothelial histology. Finally, in the setting of metastatic variant histology urethral cancer, clinicians should give consideration to systemic therapy in adenocarcinoma and other variant histology subtypes to the same extent as is done in metastatic urothelial urethral cancer, except for metastatic urethral SCC.

Our work has limitations and has to be interpreted in the context of its retrospective and population-based design. First, our cohort is based on small sample size that resulted in lack of significant differences in some of subgroup comparisons, specifically in SCC and adenocarcinoma. Second, there is a non-standardized staging and treatment pattern due to the variety of urethral cancer especially in variant histology patients. Moreover, histological classification could have been influenced by the fact that the used data derived from the SEER database without central review. Third, within other variant histology subgroup, the sample size was even smaller and required grouping of patients with several specific histological subtypes, such as Mullerian type, melanocytic, mesenchymal, sarcoma, spindle cell, and neuroendocrine. It is possible that some these subtypes were more favorable than others or vice versa (6, 15). In consequence, specific conclusions cannot be made about histological specific subtypes that were included in this subgroup. Fourth, no other information was available for patients’ performance status, comorbidities or metastatic sides, due to the design of the SEER urethral cancer database. Moreover, the administered surgical procedure could not be distinguished into more valid subcategories and also patients’ history of bladder cancer or urethral cancer bladder metastasis could have influenced treatment types and mortality rates. Additionally, differences in urethral anatomy and lack of availability of specific tumor location within the urethra have to be taken into account when our findings are interpreted. Unfortunately, the specific location of the primary could not be included in the analyses. Finally, no information was available according to the type, sequence or number of cycles of chemotherapy, which may have impact survival outcomes.

## Data Availability Statement

The raw data supporting the conclusions of this article will be made available by the authors, without undue reservation.

## Ethics Statement

Ethical review and approval was not required for the study on human participants in accordance with the local legislation and institutional requirements. Written informed consent for participation was not required for this study in accordance with the national legislation and the institutional requirements.

## Author Contributions

MW: formal analysis, methodology, investigation, writing—original draft. MD: formal analysis, methodology, investigation, writing—original draft. LN: formal analysis, investigation, writing—original draft. CC: formal analysis, investigation, writing—original draft. ZT: validation, visualization, formal analysis, methodology. SS: supervision, validation, writing—review and editing. FS: supervision, validation, writing—review and editing. ABr: supervision, validation, writing—review and editing. ABe: supervision, validation, writing—review and editing. LK: supervision, validation, writing—review and editing. FC: supervision, validation, writing—review and editing. PK: methodology, investigation, project administration, supervision, writing—original draft. All authors contributed to the article and approved the submitted version.

## Conflict of Interest

The authors declare that the research was conducted in the absence of any commercial or financial relationships that could be construed as a potential conflict of interest.
